# Editorial – *nursing open*: Introducing our new early career researcher editorial advisory board

**DOI:** 10.1002/nop2.1285

**Published:** 2022-07-20

**Authors:** Karen H. Morin, Diana‐Lyn Baptiste

**Affiliations:** ^1^ College of Nursing University of Wisconsin‐Milwaukee Milwaukee Wisconsin USA; ^2^ Johns Hopkins University School of Nursing Baltimore Maryland USA

*Nursing Open is* one of the largest open‐access nursing journals in the world, and we are extremely proud of the part we play in disseminating high‐quality nursing research and scholarship that has an impact on clinical practice globally. Our journal publishes articles on all aspects of nursing and midwifery practice, research, education and policy; contributing to the art and science of nursing. Our primary aim is to publish articles that serve as a source of knowledge and provide the best evidence for nurse researchers, nursing students, clinicians and healthcare professionals (Ali & Watson, 2016; Wiley, 2021). At *Nursing Open*, we are dedicated to promoting the dissemination of rigorous research, including those addressing timely public health issues, diversity, equity and inclusion in health care (Wiley, 2022). We regularly showcase articles that nurses and other healthcare professionals can use to inform practice improvement, influence health policy and demonstrate quality care and research. Our contributions to the science of nursing would not be the same without the dedication of our editorial team. We have an extremely skilled and experienced team of editors at *Nursing Open* (NOP) and are also supported by a diverse Editorial Board comprising eminent nursing and healthcare experts from around the world.

We are responding to feedback from *NOP* authors and reviewers telling us they would value greater involvement with the journal. In response to these matters and goals, we have taken the very exciting step of reconstituting and expanding our Early Career Researcher (ECR) Editorial Advisory Board. ECRs not only further the science of nursing but they are also the future of the profession. Our ECRs contribute to the advancement of the nursing profession by building their expertise through peer‐review and editorial activities. Given the influential role journals play in furthering nursing knowledge, helping develop ECRs to assume additional skills and roles that impact the dissemination of nursing knowledge reflects the journal’s goal of publishing cutting‐edge science while mentoring the next generation of scholars.

Using an established process, we invited persons from our reviewer base to apply for the opportunity. We were delighted with the response! And that three current ECRs re‐applied for the position. In all, we received more than 45 applications from around the world. Selecting 15 ECRs proved challenging, given the quality of the applicant pool. We selected ECRs based on the following criteria: geographical location, clinical and research expertise, and any overarching needs of the journal. As a result, our new ECR advisory board comprises international nursing scholars and clinicians from countries across the globe including Spain, Ireland, Ghana, Chile, China, Malaysia, Turkey, Poland, Australia and the USA. They bring a variety of expertise that includes nursing education, ageing, critical care, paediatrics, palliative care, implementation science, patient safety culture, mental health, epidemiology, just to name a few. We believe this diverse team will have a positive impact, maintaining the highest standards and efficiency of the peer‐review process for *NOP*.

We are looking forward to working with the new and returning ECR Editorial Advisory Board during 2022 and 2023. All editors are committed to making this experience meaningful for the ECRs. To that end, ECRS will have the opportunity not only refine their reviewing skills but also obtain feedback on reviews, thus developing as outstanding reviewers. They also will have additional opportunity to learn about other aspects associated with managing a journal.

On behalf of the Editing team at *NOP*, we look forward to working with our reconstituted ECR Editorial Advisory Board: Dr. Kate O’Reilly, Dr. Irene Aboh, Dr. Mona Alshammari, Dr. Deidre O′ Donnell, Dr. Sean Teeling, Dr. Stanley Lam, Dr. Abigail Amponash, Dr. Nina Granel‐Gimeniz, Dr. Karen Dominguez‐Cancino, Dr. Grace Cheung, Dr. Che Chong, Dr. Gulcan Eskici, Dr. Kimberly Lewis and Dr. Natalie Sak‐Dankoshy. We are so pleased to be able to provide this opportunity and look forward to learning from you on this journey. More information about our ECR Editorial Advisory Board can be found here: Nursing Open Editorial Board.

If you would like to become involved with NOP, for example, as a peer reviewer, please send an email and CV to nursingopen@wiley.com.

## CONFLICT OF INTEREST

None.
